# Therapeutic hypothermia in drowning induced hypoxic brain injury: a case report

**DOI:** 10.1186/1757-1626-2-9103

**Published:** 2009-11-27

**Authors:** Ramesh K Batra, Jonathan J Paddle

**Affiliations:** 1 Intensive Care Unit, Royal Cornwall Hospital NHS Trust, Truro, UK

## Abstract

**Background:**

Although therapeutic hypothermia for neuroprotection has been in use for over half a century but its use has been controversial in absence of proper guidelines. However for over two decades there has been revived interest in mild therapeutic hypothermia (32 - 34°C) for neuroprotection.

**Case:**

A 17 year-old female tourist was rescued from sea. She received cardio-pulmonary resuscitation for about 16 minutes. But she had sustained significant neurological insult as a result of hypoxic brain injury. Therapeutic hypothermia was added to her regime of neuroprotection in intensive care unit, and her neurological status improved in just 8 hours with full correction of her coma score by day 4.

## Introduction

Therapeutic hypothermia for neuroprotection in brain injury has been used since the 1950s. Theoretical benefits include reduction in cerebral metabolic demands, reduction in intracranial pressure, and attenuation of an array of temperature dependent deleterious biochemical processes [1]. Therapeutic hypothermia may be neuroprotective in brain injury from a number of causes. In post-hypoxic coma following cardiac arrest due to ventricular fibrillation, cooling patients to between 32°C and 34°C for 12 to 24 hours has been shown to reduce mortality and improve neurological outcome [2,3]. The neurological outcome from traumatic brain injury may also be improved with therapeutic hypothermia [4,5]. Evidence to support the use of hypothermia to manage brain injury in other conditions is weaker, but its beneficial effect has been reported in conditions such as stroke [6] and in a victim of drowning [7].

We describe the management of a patient with hypoxic brain injury post drowning, in-whom controlled therapeutic hypothermia was used.

## Case

A 17 year-old Caucasian female tourist was found floating in the sea after being caught in a strong rip and was pulled out. She was lifeless at the scene with no palpable pulse and bystander cardio-pulmonary resuscitation (CPR) was commenced immediately and continued for the next 10 minutes until the arrival of paramedics. Her Glasgow coma score (GCS) was 3, and her pupils were fixed and dilated. CPR was continued for a further 6 minutes, whilst a total of 2 mg of Epinephrine and 3 mg of Atropine were administered intravenously. This resulted in return of spontaneous circulation though with limited respiratory efforts, and an endotracheal tube was inserted to facilitate ventilation during transfer to hospital.

On arrival in the emergency department after 10 minutes by helicopter she was in sinus rhythm with heart rate of 84 beats per minute and blood pressure of 120/75 mmHg. Her neurological status remained unchanged with GCS of 3 and fixed and dilated pupils. Chest x-ray revealed bilateral pulmonary infiltrates. Initial arterial blood gases showed profound metabolic acidosis with temperature corrected pH of 6.57 (reference range [RR], 7.35 - 7.45), PCO_2 _of 7.83 kPa (RR, 4.5 - 5.5 kPa), PO_2 _of 51.9 kPa on 100% FiO_2 _and HCO_3 _of 3.6 mmol/L (RR, 22 - 26 mmol/L), base excess of -28.1 mmol/L (RR, -2 to +2 mmol/L), and lactate of 28 mmol/L (RR, <2 mmol/L). Her core temperature on admission was 33.4°C.

She was transferred to the Intensive care unit with a diagnosis of severe hypoxic brain injury and aspiration pneumonia. Conventional neuro-protective measures were commenced. This included sedation with a Propofol infusion, nursing at 15° head-up tilt with the patient's head in neutral alignment and controlled mechanical ventilation to maintain an arterial PaCO_2 _of 4.5-5 kPa. Intravenous fluids were administered to maintain a normovolaemic state and nutrition commenced via a naso-gastric tube. She was also commenced on antibiotics for the aspiration pneumonia. We decided to use therapeutic hypothermia to try and ameliorate her hypoxic brain injury. This was achieved by the insertion of the Cool-Guard endovascular cooling catheter, which was sited in her femoral vein.

**Figure 1:**
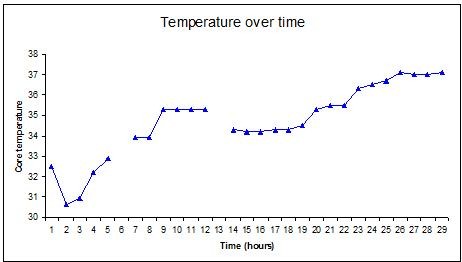
**Core temperature (°C) over time**.

**Figure 2:**
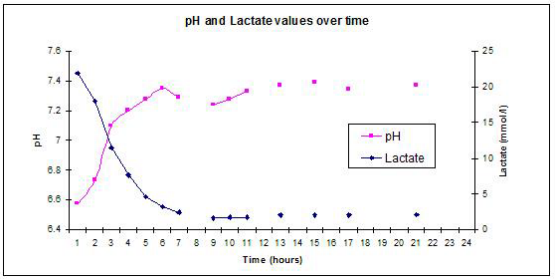
**Normalisation of arterial pH and Lactate over time**.

By the time the catheter had been sited her core temperature had dropped to 30.6°C, presumably due to redistribution of cold peripheral blood to the core, so the Cool-Guard was initially used to warm her to the target temperature of 34°C. Cooling was continued, with a target temperature of 34°C for 20 hours (Figure 1). Active cooling was stopped after 20 hours and the patient was allowed to passively re-warm. Overall, her core temperature was an average of 34.2°C for the first 24 hours of her admission. Her severe acidaemia corrected over the first 8 hours (Figure 2). Her pupils became responsive to light in 8 hours and reached normal size and reaction in 40 hours. Her GCS improved by day 3 to the point of spontaneous eye opening and obeying commands. On day 2 she suffered a seizure, confirmed by electroencephalography, which was controlled with intravenous Phenytoin (18 mg/kg). On day 4 she was transferred to a high dependency unit in France where she continued to improve to a GCS of 15. She was subsequently transferred to a rehabilitation facility. Three months post injury she was independently performing all her daily activities with no neurological deficit and the last follow-up confirmed that her discharge home was expected.

## Discussion

Therapeutic hypothermia for the specific indication of hypoxic brain injury post cardiac arrest has not been subject to a formal randomized controlled trial, and we cannot plausibly prove that hypothermia contributed to the good outcome in this case. However, clinical trials of the use of therapeutic hypothermia for other forms of brain injury, particularly brain injury post cardiac arrest [2,3], where the mechanism of neurological damage is likely to be similar, suggest that this treatment may have a role. Schaller and Graf [8] reviewed several experimental and clinical studies and concluded that neuroprotective benefit of post ischemic hypothermia directly relates to attenuation of the detrimental processes involved in both necrotic and apoptotic cell death. But there was conflicting evidence surrounding the depth and duration of hypothermia.

The International Liaison Committee for Resuscitation (ILCOR) had suggested that cooling may be beneficial for patients suffering cardiac arrest due to reasons other than ventricular fibrillation [9]. In 2002 the World Congress on Drowning in Amsterdam [10] recommended that victims of drowning who remained unconscious due to hypoxic encephalopathy should be treated with hypothermia of 32-34°C for 12-24 hours along with appropriate seizure control and continuous blood glucose monitoring.

Following ILCOR advisory statement of 2002, American heart association [11] incorporated therapeutic hypothermia in its 2005 recommendations for cardiac arrest patients. It therefore seemed reasonable to apply this intervention in our patient.

The optimum duration and degree of hypothermia remains uncertain and ILCOR recommends a temperature of 32°C to 34°C for between 12 and 24 hours [9]. There is evidence, at least in traumatic brain injury (5), that more prolonged periods of hypothermia may have greater benefit. A retrospective review of hypothermia for the management of drowning in 40 children[12] found a worse outcome for patients treated with hypothermia compared to normothermic patients when both the duration (24 to 36 hours) and degree of cooling (30°C to 33°C) was greater than in our case. The patients in this series were also treated with hyperventilation and high-dose barbiturates.

Williamson and colleagues[7] reported a successful outcome in a victim of drowning treated with therapeutic hypothermia. Their case shares many similarities with ours, including profound metabolic acidosis, hyperlactaemia and low GCS with fixed, dilated pupils at presentation. The authors concluded that, in the context of a victim of drowning and in association with hypothermia, these signs may not carry the grave prognosis they would otherwise in critically ill patients. Our findings in this case support this view.

Our experience supports the role of therapeutic hypothermia as part of the management strategy of patients who have suffered hypoxic brain injury as a result of drowning.

## Consent

As is evident from the case report the anonymity of the patient is preserved in every aspect to maintain confidentiality and privacy of the patient.

The report states that the patient stayed in our institution for 4 days only, during which time we had not decided to publish the case report. After repatriation of the patient much effort was taken to get the consent from the patient and her parents, but due to continuous movement of the patient from the hospital to the rehabilitation facility it became extremely difficult, the language bar also posed a real challenge towards the consent obtaining procedure

## Conflicts of interests

The authors declare that they have no competing interests.

## Authors' contributions

JJP was the attending intensivist and anaesthetist for the patient. He included therapeutic hypothermia in the plan of management of the patient. RB collected and assimilated the data towards formulation of the case report.
